# Regulation of the methanogenesis pathways by hydrogen at transcriptomic level in time

**DOI:** 10.1007/s00253-023-12700-3

**Published:** 2023-08-23

**Authors:** Márk Szuhaj, Balázs Kakuk, Roland Wirth, Gábor Rákhely, Kornél Lajos Kovács, Zoltán Bagi

**Affiliations:** 1 Department of Biotechnology, University of Szeged, Szeged, Hungary; 2 Department of Medical Biology, University of Szeged, Szeged, Hungary; 3 Biological Research Center, Institute of Plant Biology, Szeged, Hungary; 4 Biological Research Center, Institute of Biophysics, Szeged, Hungary

**Keywords:** Hydrogenotrophic methanogens, Hydrogen, Methanogenesis, Power to gas, Redox regulation

## Abstract

**Abstract:**

The biomethane formation from 4 H_2_ + CO_2_ by pure cultures of two methanogens, *Methanocaldococcus fervens* and *Methanobacterium thermophilum*, has been studied. The goal of the study was to understand the regulation of the enzymatic steps associated with biomethane biosynthesis by H_2_, using metagenomic, pan-genomic, and transcriptomic approaches. Methanogenesis in the autotrophic methanogen *M. fervens* could be easily “switched off” and “switched on” by H_2_/CO_2_ within about an hour. In contrast, the heterotrophic methanogen *M. thermophilum* was practically insensitive to the addition of the H_2_/CO_2_ trigger although this methanogen also converted H_2_/CO_2_ to CH_4_. From practical points of view, the regulatory function of H_2_/CO_2_ suggests that in the power-to-gas (P2G) renewable excess electricity conversion and storage systems, the composition of the biomethane-generating methanogenic community is essential for sustainable operation. In addition to managing the specific hydrogenotrophic methanogenesis biochemistry, H_2_/CO_2_ affected several, apparently unrelated, metabolic pathways. The redox-regulated overall biochemistry and symbiotic relationships in the methanogenic communities should be explored in order to make the P2G technology more efficient.

**Key points:**

*• Hydrogenotrophic methanogens may respond distinctly to H*_*2*_ */CO*_*2*_ *in bio-CH*_*4*_ *formation.*

*• H*_*2*_ */CO*_*2*_ *can also activate metabolic routes, which are apparently unrelated to methanogenesis.*

*• Sustainable conversion of the fluctuating renewable electricity to bio-CH*_*4*_ *is an option.*

## Introduction

Anaerobic digestion (AD) is one of the most promising technologies among the renewable and sustainable bioenergy production processes, offering multiple benefits such as waste and by-product biomass utilization, production of gaseous biofuel, and organic fertilizer (Bagi et al. 2017). Biogenic methane production is a complex microbial process carried out by a unique community of bacteria and archaea. The methanogenic archaea are among the main contributors to the global carbon cycle and exclusively belong to the Euryarchaea phylum (Berghuis et al. 2019). Methanogenesis is an anaerobic respiration process that uses oxidized carbon such as CO_2_ as a terminal electron acceptor (Lyu et al. 2018). The methanogenic archaea are a highly specialized group of microbes as they produce CH_4_, which is a useful energy carrier when utilized and a powerful greenhouse gas when released. Although methanogens utilize only a limited number of simple substrates, their biochemistry is rather complex and unconventional in the microbial world. Three major pathways of methanogenesis are known: hydrogenotrophic, methylotrophic, and acetoclastic (Conrad 2009; Zhao et al. 2018). The hydrogenotrophic methanogens convert H_2_ and CO_2_ to CH_4_, and the acetoclastic methanogens split acetate to CH_4_ and CO_2_, while methylotrophic methanogens use methylated compounds for methane production.

Biochemical methane evolution requires at least six unusual coenzymes, some of which are active only at extremely low redox potentials (Lyu et al. 2018). The only enzyme present in all types of methanogenesis is methyl-coenzyme M reductase (Mcr), a Ni-corrinoid protein catalyzing the last step of methyl group reduction to methane (Liu and Whitman 2008). In hydrogenotrophic methanogenesis, the regulatory roles of the local H_2_ levels and interspecies H_2_ transfer have been recognized as a central element in the concerted action of the complex microbial community (Bagi et al. 2007; Giovannini et al. 2016; Sunyoto et al. 2016). H_2_ serves as a delicate safety valve: when H_2_ accumulates, it creates product inhibition of the acetogenic bacteria, which are the main H_2_ producers in the AD community (Ahring and Westermann 1988).

At the same time, hydrogenotrophic methanogens require as much H_2_ as the system can provide, because they use H_2_ for the CO_2_ reduction to CH_4_. The competition and sustainable equilibrium between H_2_ production by acetogens and H_2_ consumption by hydrogenotrophic methanogens usually result in a very low dissolved H_2_ partial pressure in order to maintain a balanced operation of the entire microbiological community (Vavilin et al. 1995). The extremely low solubility of H_2_ in the aqueous environment is a rate-limiting factor. Consequently, the hydrogenotrophic methanogens starve for H_2_ in the AD processes, and thus, the H_2_ production represents a bottleneck in the biomethane formation (Demirel and Scherer 2008; Kern et al. 2016; Szuhaj et al. 2016).

We demonstrated earlier that reduced accessibility is a regulating element in biogas production and corroborated that the introduction of H_2_-producing bacteria into a natural biogas-generating consortium appreciably increased the efficacy of biogas production both in batch fermentations and in scaled-up anaerobic digestion (Bagi et al. 2007). The relationship between the acetogens and methanogens is syntrophic, supported by a process called interspecies hydrogen transfer or interspecies electron flow (Rotaru et al. 2014). The actual H_2_ concentration has been shown to determine the composition of the methanogenic community (Ács et al. 2019).

The expression/transcription of genes in hydrogenotrophic methanogens has been reported to change in response to the H_2_ availability, and H_2_ alters the physiology of several microbes belonging to the kingdom Bacteria (Kakuk et al. 2021). Hence, the regulatory role of H_2_ on the expression of methanogenesis genes is instinctively expected. H_2_-regulated expression of genes, which are apparently not directly involved in H_2_ metabolism, allows the exploration of the underlying metabolic connections and improves our knowledge concerning molecular redox networks in the biogas-producing microbial community and in environmental microbiology in general.

A rapidly emerging biotechnological application of the H_2_ metabolism of hydrogenotrophic methanogens is the power-to-gas (P2G) system development. This innovative approach employs the ability of hydrogenotrophic methanogens to efficiently reduce CO_2_ using the reducing power of H_2_ generated from renewable electricity via water electrolysis (Szuhaj et al. 2016; De Corato et al. 2022; Pastore et al. 2022). Renewable electricity, produced in a fluctuating manner by the photovoltaic and/or wind energy power plants, can be converted to the easily storable and transportable biomethane energy carrier and introduced into the natural gas pipelines. The benefits and advantages of this energy conversion and storage concept have been discussed recently (Bassani et al. 2016; Angelidaki et al. 2018; Palù et al. 2022). The biological conversion of electrolytically generated reductants to CH_4_ is appealing also as a tool to reduce the carbon footprint imposed upon the Earth by fossil energy–fueled human activities (Yan et al. 2021; Zhang et al. 2022). Biomethanation can be carried out by pure cultures of hydrogenotrophic methanogens or mixed anaerobic microbial communities containing hydrogenotrophic methanogens. The methanogens are more stable in a mixed culture system, and the operational costs are lower, although the molecular events can be followed clearly in pure cultures. The dynamics of the “turn-on” response of the hydrogenotrophic methanogens to the swiftly changing H_2_ supply, which is due to the unpredictable H_2_ generation by the weather-dependent renewable electricity production, is not fully explored yet.

It is astonishing to note the complexity of the molecular machinery, which handles the simplest molecule, H_2_. The aims of the present study have been to determine the expression levels of the genes involved in hydrogenotrophic methanogenesis and to map the expression profile changes in hydrogenotrophic methanogens during the “turn-on” and “turn-off” phases of P2G conversion.

## Materials and methods

### Strains and media

Strains were obtained from DSMZ (Leibniz Institute DSMZ-German Collection of Microorganisms and Cell Cultures GmbH, Braunschweig). *Methanocaldococcus fervens* (DSM 4213) was isolated from a deep-sea hydrothermal vent sample from Guaymas Basin, Gulf of California (Jeanthon 1999). Growth occurs between 48 and 92 °C, with an optimum of around 85 °C, and between pH 5.5 and 7.6, with an optimum of about pH 6.5. It is a chemolithotrophic strain that uses H_2_ and CO_2_ as energy and carbon sources to produce methane. *Methanobacterium thermophilum* (DSM 6529) grows between 40 and 70 °C, with an optimum at 55 °C. *M. thermophilum* reduces CO_2_ with H_2_ but also requires formate and/or acetate for growth (Kotelnikova et al. 1993).

*M. fervens* and *M. thermophilum* were incubated in DSM 282 medium, which initially consisted of 0.14 g L^−1^ K_2_HPO_4_; 0.14 g L^−1^ CaCl_2_ × 2 H_2_O; 0.25 g L^−1^ NH_4_Cl; 3.4 g L^−1^ MgSO_4_ × 7 H_2_O; 4.1 g L^−1^ MgCl_2_ × 6 H_2_O; 0.33 g L^−1^ KCl; 0.5% (v v^−1^) NiCl_2_ × 6 H_2_O solution (0.1% w v^−1^); 30 g L^−1^ NaCl; 0.1 g L^−1^ Fe(NH_4_)_2_(SO_4_)_2_ × 6 H_2_O; trace element solution (DMS 141); 0.5% v v^−1^ Na-resazurin solution (0.1% w v ^−1^); 1 g L^−1^ NaHCO_3_; 1% v v^−1^ vitamin solution (DSM 141); 0.5 g L^−1^ L-cysteine-HCl × H_2_O; and 0.5 g L^−1^ Na_2_S × 9 H_2_O. The trace element solution (DSM 141) contained 2 g L^−1^ biotin; 2 g L^−1^ folic acid; 10 g L^−1^ pyridoxine hydrochloride; 5 g L^−1^ thiamine hydrochloride; 5 g L^−1^ riboflavin hydrochloride; 5.0 g L^−1^ nicotinic acid; 5 g L^−1^ DL-calcium pantothenate; 0.1 g L^−1^ vitamin B12; 5 g L^−1^ *p*-aminobenzoic acid; and 5 g L^−1^ lipoic acid. The vitamin solution (DSM 141) contained 1.5 g L^−1^ nitrilotriacetic acid; 3 g L^−1^ MgSO_4_ × 7 H_2_O; 0.5 g L^−1^ MnSO_4_ × H_2_O; 1 g L^−1^ NaCl; 0.1 g L^−1^ FeSO_4_ × 7 H_2_O; 0.18 g L^−1^ CoSO_4_ × 7 H_2_O; 0.1 g L^−1^ CaCl_2_ × 2 H_2_O; 0.18 g L^−1^ ZnSO_4_ × 7 H_2_O; 0.01 g L^−1^ CuSO_4_ × 5 H_2_O; 0.02 g L^−1^ KAl(SO_4_)_2_ × 12 H_2_O; 0.01 g L^−1^ H_3_BO_3_; 0.01 g L^−1^ Na_2_MoO_4_ × 2 H_2_O; 0.03 g L^−1^ NiCl_2_ × 6 H_2_O; 0.3 mg L^−1^ Na_2_SeO_3_ × 5 H_2_O; and 0.3 mg L^−1^ Na_2_WO_4_ × 2 H_2_O. The pH of the completed medium was adjusted to 7–7.2. The headspaces of the reactors were replaced by a sterile 80% H_2_ and 20% CO_2_ gas mixture. The pure cultures have been maintained in anaerobic serum bottles.

### Fermentation system

The reaction vessels simulate a CSTR (continuous-stirred tank reactor) system, using Braun CT5-2 (B. Braun Biotech International GmBH., Melsungen, Germany) bioreactors with a total volume of 5 L and 2 L of headspace. The sterile media were inoculated with methanogen cultures in 1 V/V%. The sealed headspaces of the reactors were replaced by flushing with a sterile gas mixture of 80% H_2_ and 20% CO_2_ from a gas cylinder (Linde, Dublin, Ireland; 5 min, 2.5 L min^−1^). The incubation temperature of the reactors was 55 °C (*M. thermophilum*) and 85 °C (*M. fervens*), respectively. Methanogens were cultivated until OD_600nm_ = 0.15–0.20. In the case of *M. fervens*, it required 1 day, and in the case of *M. thermophilum*, 4 days were needed to achieve this cell density. When the expected optical density was reached, the first samples (150 mL) were taken for transcriptomic analysis, and these were marked as Metfer_H_2__start and Metthe_H_2__start, respectively (see figures). The headspace was then replaced with sterile N_2_ (Linde 4.5, 5 min, 2.5 L min^−1^) for 1 h, and these samples were entitled Metfer_N_2_. In order to examine the response of the heterotrophic *M. thermophilum* to the withdrawal of H_2_/CO_2_, Na-acetate (1 g L^−1^) and Na-formate (3 g L^−1^) were injected into the corresponding reactors. Metthe_N_2_ + VFA sampling took place after 1 h following the H_2_ supply “turn-off” event. Afterward, the headspaces of the reactors were readjusted to H_2_/CO_2_ (Linde, 5 min, 2.5 L min^−1^). The last samples were taken after an additional 1-h incubation, these were labeled “Metfer_H_2__end” and “Metthe_H_2_ + VFA_end.” The whole procedure was repeated three times, and the transcriptomic analysis processed the data of three biological parallels. Samples taken from the three parallel experiments were pooled for RNA isolation and sequencing.

### Total RNA isolation and transcriptome sequencing

The RNA extractions were carried out with the Zymo Research Soil/Fecal RNA kit (R2040, Zymo Research, Irvine, CA, USA). After lysis (bead beating), the Zymo Research kit protocol was followed. The DNA contamination was removed by Thermo Scientific Rapid Out™ DNA removal kit (K2981, Thermo Fisher Scientific, Waltham, MA, USA). Before transcriptome sequencing, rRNA was depleted from RNA by using the Gram + /Gram − depletion kit in a 60:40 ratio (RiboMinus A15020 Life Technologies, San Francisco, CA, USA). The mRNA library was prepared using the mRNA Sample Prep kit (Illumina, San Diego, CA, USA). Sequencing was performed using the Illumina V2 chemistry (2 × 250 bp) and applying the MiSeq paired-end mode. Raw sequences are available on the NCBI Sequence Read Archive (SRA) under the accession number: PRJNA922065.

### Pangenome construction and mapping of transcript data on gene clusters

The genome of *M. thermophilum* and *M. fervens* was downloaded from the NCBI Genome database. The Anvi’o (v7 “Hope”) (Eren et al. 2015) pan-genomics workflow was used in computing the archaeal pangenome. In the first step, genome databases were generated where DNA and amino acid sequences, functional annotations (based on InterPro and COG (clusters of orthology groups of proteins) databases), and databases of each gene in genomes were stored. In the second step, pangenome was built up in the following way: (1) calculation of amino acid similarities between the genomes using blastp, (2) identification of gene clusters with MCL (Markov Cluster) algorithm, (3) computation of a total number of genes, gene clusters across genomes, and core clusters that appear in both genomes, and (4) hierarchical clustering analysis for gene clusters and genomes using the Euclidean distance and the Ward clustering. In this step, transcriptomic data were used to create a “pan-genomics” analysis based on (Delmont and Eren 2018) where the average coverage of genes in gene clusters across genomes was recovered. In the final step, pangenome was visualized by Anvi’o interactive interface. For measuring the change in the expression level of genes (log_2_ fold change), CLC Genomics Workbench (v20) was employed (log2 fold change calculation).

## Results

**Fig. 1:**
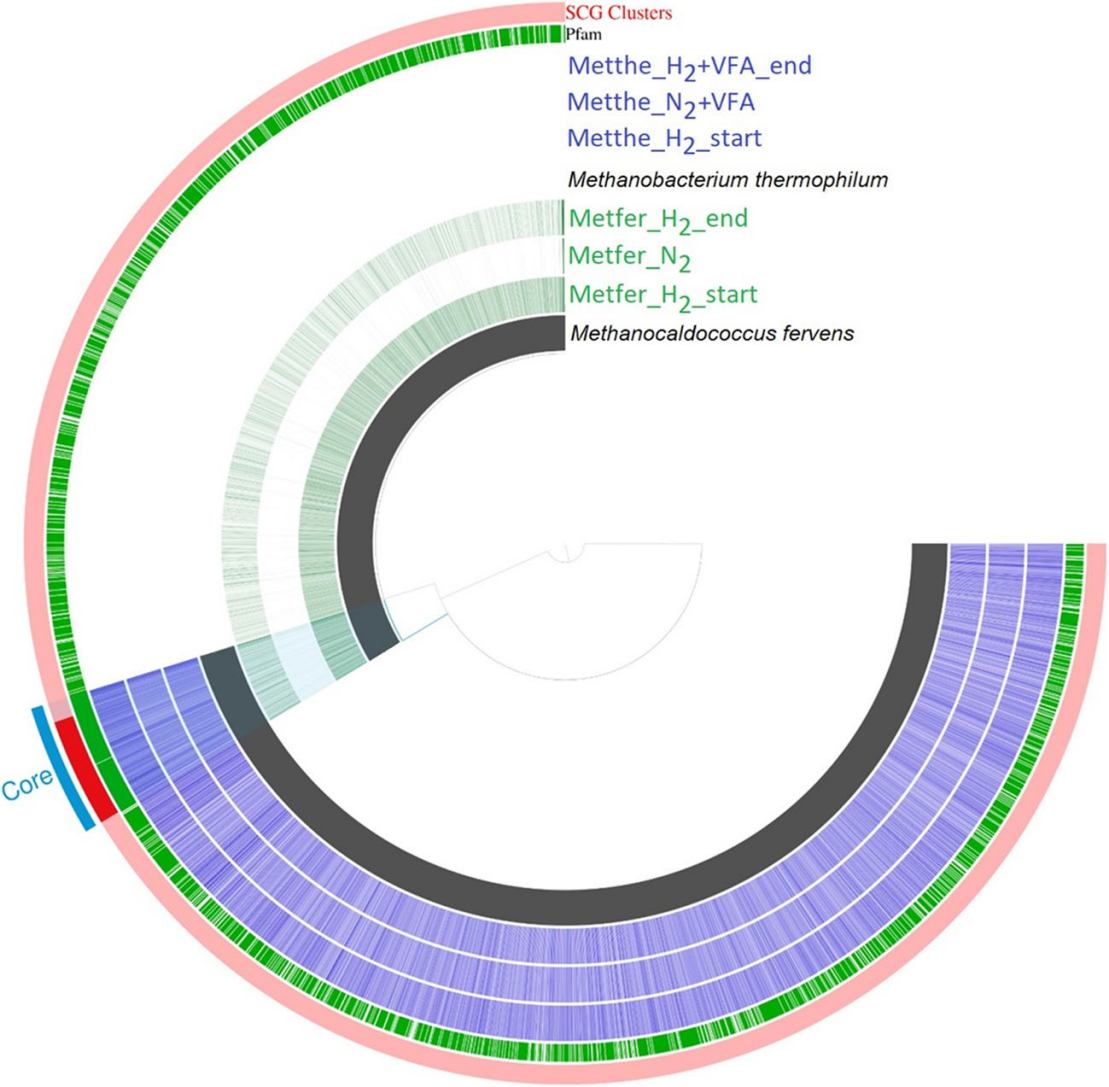
The pangenome and metatranscriptome of *M. fervens* (Metfer) and *M. thermophilum* (Metthe). The innermost ring shows the total genome of *M. fervens* (black). The next 3 layers represent the coverage of the genes which were annotated in the genome (the color darkness increases with the gene coverages) at the 3 sampling points: Metfer_H_2__start, Metfer_N_2_, and Metfer_H_2__end. The 4th ring represents the total genome of *M. thermophilum* (black). The next 3 layers represent the coverage of the genes which were annotated in the genome (the color darkness increases with the gene coverages) at the 3 sampling points: Metthe_H_2__start, Metthe_N_2_ + VFA, and Metthe_H_2_ + VFA_end. The green ring indicates the gene clusters in which at least one gene was functionally annotated using Pfams. In the outermost ring, the red bands represent the distribution of SCG clusters (genes, that are present in both organisms and overlap) of the two archaea. The blue band marks the position of core hydrogenotrophic methanogenesis genes in the two genomes

*M. fervens*, being an obligate hydrogenotrophic methanogen, showed active expression of the core and the accessory genes (Fig. 1: Metfer_H_2__start) in the H_2_/CO_2_-rich environment. The core genes, which are directly involved in hydrogenotrophic methanogenesis, were highly expressed upon supplying H_2_/CO_2_, relative to the accessory genes (see the blue line marked “core” in Fig. 1). When the headspaces of the reactors were replaced by N_2_ for 1 h, the expression profiles changed relative to the H_2_/CO_2_ that supported hydrogenotrophic metabolism (Fig. 1 Metfer_N_2_). Upon replacing the gas phase again with the initial H_2_/CO_2_, the transcriptomic activity of *M. fervens* was recovered (Metfer_H_2__end). The expression of the genes after 1 h reached almost the starting level.

**Fig. 2:**
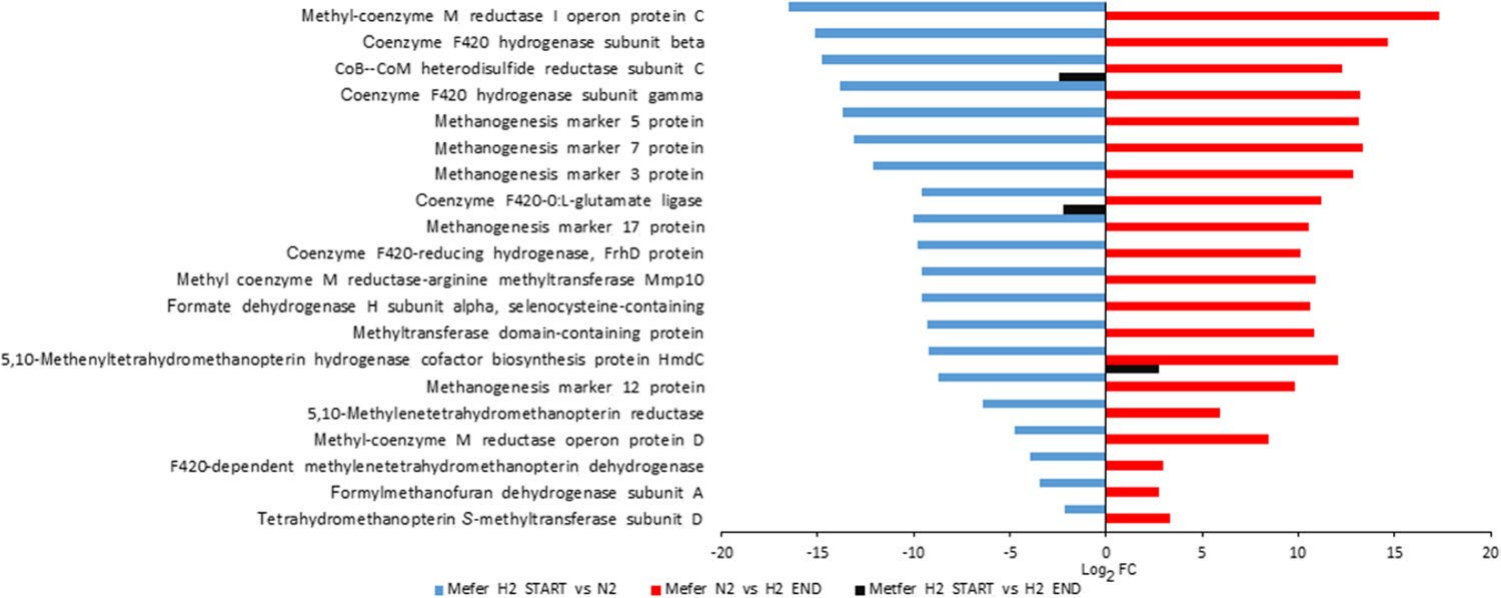
Significant (− 2 ≥ log_2_FC ≤ 2) gene expression changes in the core transcriptome of *M. fervens* (Metfer). Blue bars: expression changes between H_2_/CO_2_ versus N_2_ supplied (Metfer_H_2__start and Metfer_N_2_ samples). Red bars: expression changes between Metfer_N_2_ and Metfer_H_2__end samples. Black bars: expression changes between Metfer_H_2__start and Metfer_H_2__end samples. ${\mathrm{log}}_{2}\mathrm{FC}={\mathrm{log}}_{2}\left(\frac{\mathrm{relative\;abundance\;sample\;}1}{\mathrm{relative\;abundance\;sample\;}2}\right)$

It is noteworthy, that the genome-wide transcript profile clearly indicated the negative effect of the H_2_/CO_2_ withdrawal on the expressions of the core methanogenesis and the accessory genes in *M. fervens*. The most pronounced changes were apparent among the methanogenesis-related genes (Fig. 2). Some of these genes code for methanogenesis marker proteins, such as those playing an important role in the functional activity of the hydrogenotrophic methane formation, i.e., methylene-tetrahydromethanopterin (methylene-H_4_MPT) reductase, -hydrogenase, coenzyme F_420_ reducing hydrogenase, F_420_ hydrogenase, CoB-CoM heterodisulfide reductase, F_420_-dependent methylene-H_4_MPT dehydrogenase, formate dehydrogenase, formylmethanofuran dehydrogenase, methyl-coenzyme M reductase, and tetrahydromethanopterin *S*-methyltransferase. Correlation between the scale of alteration in the biological activity of several genes in response to H_2_/CO_2_ “switch on” and “switch off” could be observed, see blue and red horizontal columns in Fig. 2. The restoration of the methanogenic activity at the transcriptomic level corroborates rapid H_2_-driven response, which is shorter than 1 h in this system, in the obligate autotrophic *M. fervens* (Figs. 1 and 2).

**Fig. 3:**
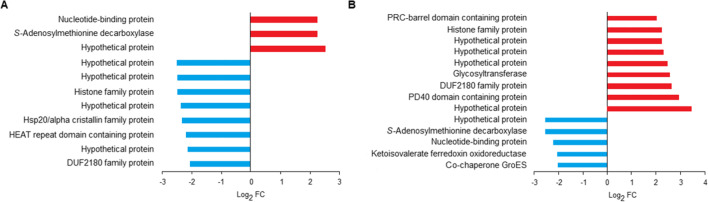
Significant (− 2 ≥ log_2_FC ≤ 2) gene expression changes in the core transcriptome of *M. thermophilum* (Metthe). **A** Negative (blue bars) and positive (red bars) expression changes between Metthe_H_2__start and Metthe_N_2_ + VFA samples. **B** Negative (blue bars) and positive (red bars) expression changes between Metthe_N_2_ + VFA and Metthe_H_2_ + VFA_end samples.${\mathrm{log}}_{2}\mathrm{FC}={\mathrm{log}}_{2}\left(\frac{\mathrm{relative\;abundance\;sample\;}1}{\mathrm{relative\; abundance\;sample\;}2}\right)$

The metabolism of *M. thermophilum* differs from that of *M. fervens*. *M. thermophilum* is also a hydrogenotrophic archaeon able to utilize H_2_ for CO_2_ reduction but needs formate/acetate (volatile fatty acids (VFA)) in its heterotrophic lifestyle (Rivard and Smith 1982; Maestrojuan et al. 1990). The injected VFA sustained the apparent biological activity of the core and accessory genes. Thus, significant changes did not appear upon “H_2_ starvation.” VFA, the alternative methanogenesis substrate, compensated for the H_2_ absence in the hydrogenotrophic metabolism; therefore, the methanogenesis-related genes remained highly expressed (Fig. 1). Nevertheless, the detailed analysis of the core gene expressions revealed significant transcriptional changes (Fig. 3).

It is noted at first glance that differential expression and biological activity of the hydrogenotrophic methanogenesis genes were not evident in *M. thermophilum*. Heterotrophic methanogenesis took over after the withdrawal of the H_2_/CO_2_ supply; hence, the methanogenesis-related genes were still expressed and ready to perform the expected biological activity. That is why in the differential expression profiles the genes coding for key enzymes of hydrogenotrophic metabolism did not emerge (Fig. 3). The removal of the H_2_/CO_2_ and concomitant addition of VFA increased the expression of a nucleotide-binding protein and the *S*-adenosylmethionine decarboxylase. *S*-Adenosylmethionine decarboxylase is a polyamine that functions as an activated methyl donor for cells to modify RNA, DNA, proteins, lipids, and cofactors and has an alternate function in spermidine biosynthesis (Kim et al. 2000). The change of the gas supply significantly decreased the expression of several hypothetical proteins, such as the domain of unknown function (DUF) 2180 protein, histone family protein, heat repeat domain–containing protein, and Hsp20/alpha crystallin family protein. Most of these genes code for proteins of unknown or poorly understood functions. Heat shock proteins take part in the protection against environmental stresses. They act as chaperons, protecting target proteins from denaturation, aggregation, and inactivation. Reversing the supply of H_2_/CO_2_ elevated the expression of the genes coding for unknown proteins, protein domains (hypothetical proteins, DUF 2180 protein, PD40 domain–containing protein, PRC-barrel domain-containing protein, histone family protein, and glycosyltransferase. The transient elevated partial pressure of H_2_/CO_2_ inhibited the expression of ketoisovalerate ferredoxin oxidoreductase and *S*-adenosylmethionine decarboxylase. Ketoisovalerate ferredoxin oxidoreductase catalyzes the coenzyme A-dependent oxidation of branched-chain 2-ketoacids coupled to the reduction of ferredoxin (Heider et al. 1996).

The investigation of the mRNA-predicted COGs (clusters of orthology groups of proteins) helps to track the changes of functionally connected gene groups. The COG analysis correlated with the expression profiles of the whole genome (Fig. 1).

**Fig. 4:**
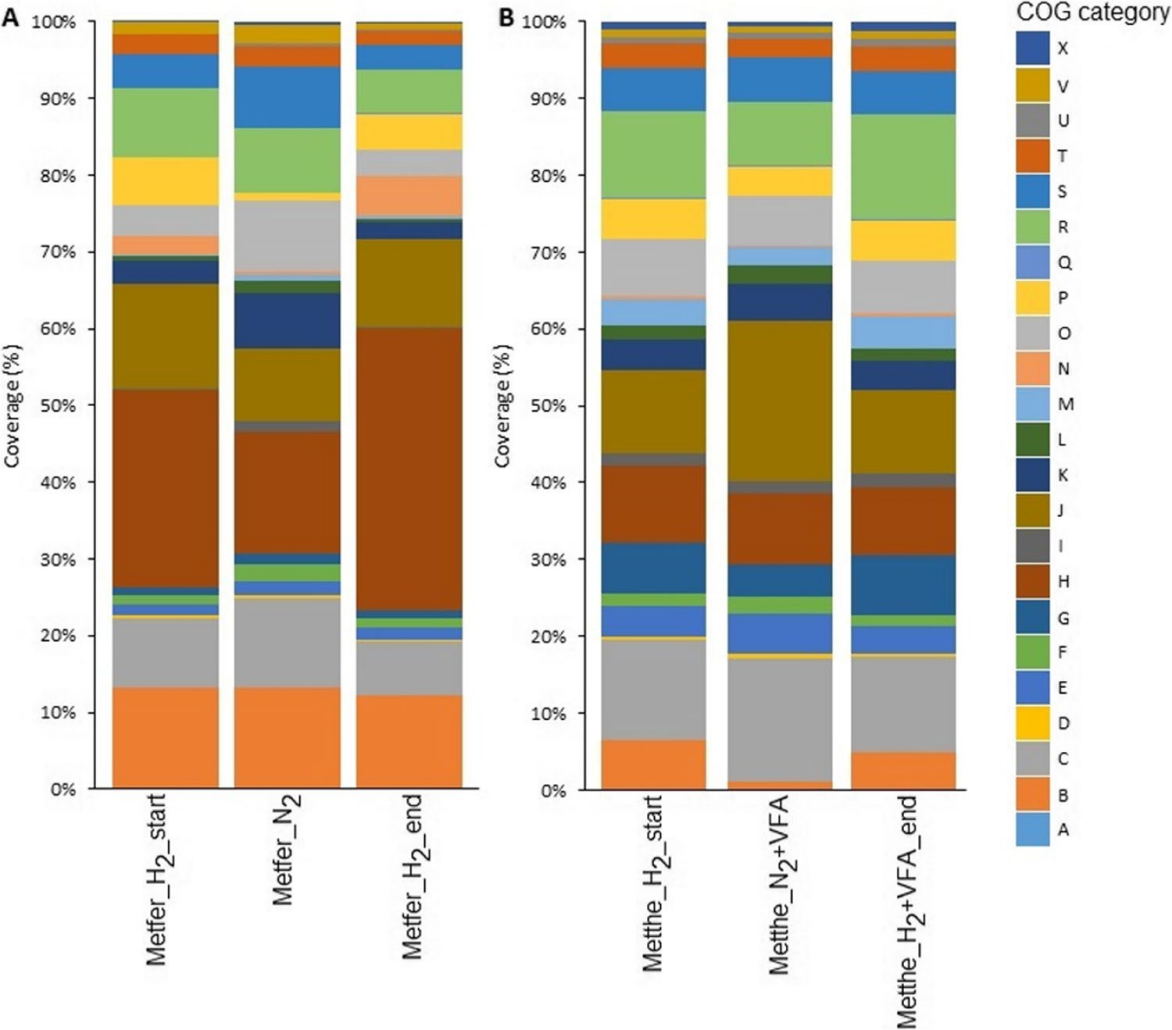
Transcriptomic changes in the COG categories due to the presence and absence of reductants (H_2_ or H_2_ + VFA) in *M. fervens* (Metfer) and *M. thermophilum* (Metthe), respectively. The coverage % values represent the coverage of the COGs relative to the total genes annotated in the genomes

Despite the distinct fermentation environments, the COGs’ changes were similar in both organisms (Fig. 4).

The most spectacular change was the decrease in the expression of category H genes in response to H_2_/CO_2_ removal in *M. fervens*. The coenzyme transport and metabolism genes (COG H) expressed the majority of the annotated sequences under H_2_/CO_2_ (25.66% in Metfer_H_2__start) in *M. fervens*, which decreased to 15.98% upon diminishing H_2_/CO_2_. (Metfer_N_2_) After restoring H_2_/CO_2_ feeding, the expression of the COG group H genes was quickly reactivated, and the gene activity of the COG group H surpassed the previously detected level (36.64%, Metfer_H_2__end).

The transcriptomic changes of the genes coding for coenzyme transport and metabolism (COG H) group were less dominant in *M. thermophilum* during similar treatment. These genes were similarly abundant (10.08%) as those belonging in category C (energy production and conversion, 13.14%), K (transcription, 10.85%), and R (predicted general functions, 11.22) COGs. Following the H_2_ gas supply switch-off, no remarkable changes were detected presumably due to the activation of the heterotrophic methanogenic pathway via the organic substrate addition. Restoring the H_2_/CO_2_ supply did not increase the transcriptomic activity of the genes in these categories.

COG category C harbors most of the core genes of hydrogenotrophic methanogenesis. As opposed to the H category, expression of genes in category C remained stable throughout the entire fermentation, the H_2_/CO_2_ withdrawal slightly elevated the relative read coverage of these genes from 9.03 to 11.56% in *M. fervens* and from 13.14 to 16.13% in *M. thermophilum*, respectively. Surprisingly, COG category J genes showed high expression upon H_2_/CO_2_ withdrawal in *M. thermophilum*, which might be due to the obligatory addition of VFA to sustain *M. thermophilum*, rather than displaying an effect of changing gas phase composition. Category J genes code for translation, ribosomal structure, and biogenesis; hence, they may not be directly linked with hydrogenotrophic methanogenesis.

No hydrogenotrophic methanogenesis-related significant (− 2 ≥ log_2_FC ≤ 2) COG changes occurred in *M. fervens* in I (lipid transport and metabolism), N (cell motility), P (inorganic ion transport and metabolism), U (intracellular trafficking, secretion, and vesicular transport), and X (nuclear structure) and in *M. thermophilum* in B (chromatin structure and dynamics). These COGs are therefore not directly connected with the hydrogenotrophic metabolism in these Archaea.

## Discussion

Our aim in this study was to determine the transcriptional activity of two thermophilic hydrogenotrophic methanogens (*M. fervens*, *M. thermophilum*) in response to H_2_/CO_2_ “turn-on” and “turn-off” conditions. The two strains utilize somewhat distinct methanogenesis pathways in the presence of various carbon sources (Ferry 2011). H_2_ is oxidized, and CO_2_ is reduced to CH_4_ in the obligate auxotrophic hydrogenotrophic CO_2_-reduction pathway, e.g., in *M. fervens* (Jeanthon 1999). Both strains are able to reduce CO_2_ via the auxotrophic pathway, but *M. thermophilum* also requires formate and/or acetate for growth and operates according to facultative hydrogenotrophic methanogenesis (Narihiro et al. 2016).

Pan-genomics-based transcriptomics provides detailed information about core and accessory genes in closely related genomes (Delmont and Eren 2018). The results uncover the transcriptomic activity of specific strains, which tell us how actively the functions of the particular genes, or clusters thereof, have worked at the time of sampling. The H_2_/CO_2_ removal by replacing the dissolved gases with inert N_2_ gas predestined a shift in the hydrogenotrophic gene expression and the alteration of the methanogenic metabolism in both studied hydrogenotrophic organisms. The questions were as follows: which genes were turned on and off upon this intervention and what was the time duration needed for changing the on–off switch position? What were the conceivable effects of the observed pan-genomic/pan-transcriptomic changes from the point of view of an operating P2G system?

A fundamental difference in gene expression profiles between obligate autotrophic (*M. fervens*) and facultative heterotrophic (*M. thermophilum*) methanogenesis was displayed (Fig. 1). Taking this difference into account, the same experimental protocol was applied for both strains. The total transcript changes indicated rapid inhibition of obligate autotrophic methanogenesis in *M. fervens* upon the diminishing H_2_/CO_2_ supply. The majority of the genes in the genome were apparently also affected, indicating a general reorganization of the genome expression profile. *M. fervens* was able to rapidly restore its expressional activity after replenishing H_2_/CO_2_. This observation is interpreted as a relatively quick reorganization of interconnected and apparently unrelated gene expression networks. In contrast, the hydrogenotrophic methanogenesis in *M. thermophilum* did not go through similar remarkable gene expression change. The available organic VFA kept the hydrogenotrophic gene expression at a stable level in the absence of H_2_/CO_2_. In the power-to-gas applications, *M. fervens* would be more suitable than *M. thermophilum*, although using the mixed anaerobic methanogen communities for the P2G operation seems the best choice based on economical considerations and operational stability.

This study also demonstrated that almost the entire metabolism of hydrogenotrophic methanogens is affected by the presence or absence of H_2_/CO_2_. The present knowledge does not allow drawing any specific conclusion about the contribution of the hitherto largely unknown metabolic pathways to the hydrogenotrophic methanogenesis. Nevertheless, these observations demand further systematic studies. The distant metabolic routes are presumably coupled to the intracellular redox regulation in hydrogenotrophic archaea.

Among the COG categories, the genes belonging to categories C and H deserve special attention. The genes in the COG category C are responsible for energy production and conversion (Tatusov et al. 2001), and together with the genes in the COG category H, they are the most highly expressed groups in methanogens (Gilmore et al. 2017). Formylmethanofuran dehydrogenase, coenzyme F_420_-reducing hydrogenase/dehydrogenase, and F_420_-dependent methylene-tetrahydromethanopterin dehydrogenase (Mtd) core hydrogenotrophic methanogenic genes of COG category C were annotated in the two archaea.

Formylmethanofuran dehydrogenase is the first enzyme of the hydrogenotrophic methanogenesis pathway. This enzyme catalyzes the binding of CO_2_ to the amino group of methanofuran, forming formylmethanofuran (Bertram et al. 1994). Coenzyme F_420_-reducing hydrogenase is responsible for the formation of reduced coenzyme F_420_ (F_420_H_2_), which is the electron donor for the methenyl-tetrahydromethanopterin (methenyl-H_4_MPT) reduction. Additionally, the F_420_H_2_ is one of the electron sources for the F_420_ H_2_-dependent methylene-tetrahydromethanopterin dehydrogenase (Mtd) (Hendrickson and Leigh 2008). Tetrahydromethanopterin *S*-methyltransferase (Mtr) is a membrane-associated enzyme complex, which catalyzes a Na^+^ translocation-dependent methyl transfer from methyl-tetrahydromethanopterin (methyl-H_4_MPT) to coenzyme M (CoM-SH), creating methyl-coenzyme M (CH_3_-S-CoM). Methyl-coenzyme M (CH_3_-S-CoM) is a precursor molecule of the heterodisulfide complex (CoB-S–S-CoM) formation. Methyl-coenzyme M reductase (Mcr) catalyzes the reduction of methyl-coenzyme M (CH_3_-S-CoM) with coenzyme B (CoB-SH) (Wagner et al. 2018) Fig. 5.

**Fig. 5:**
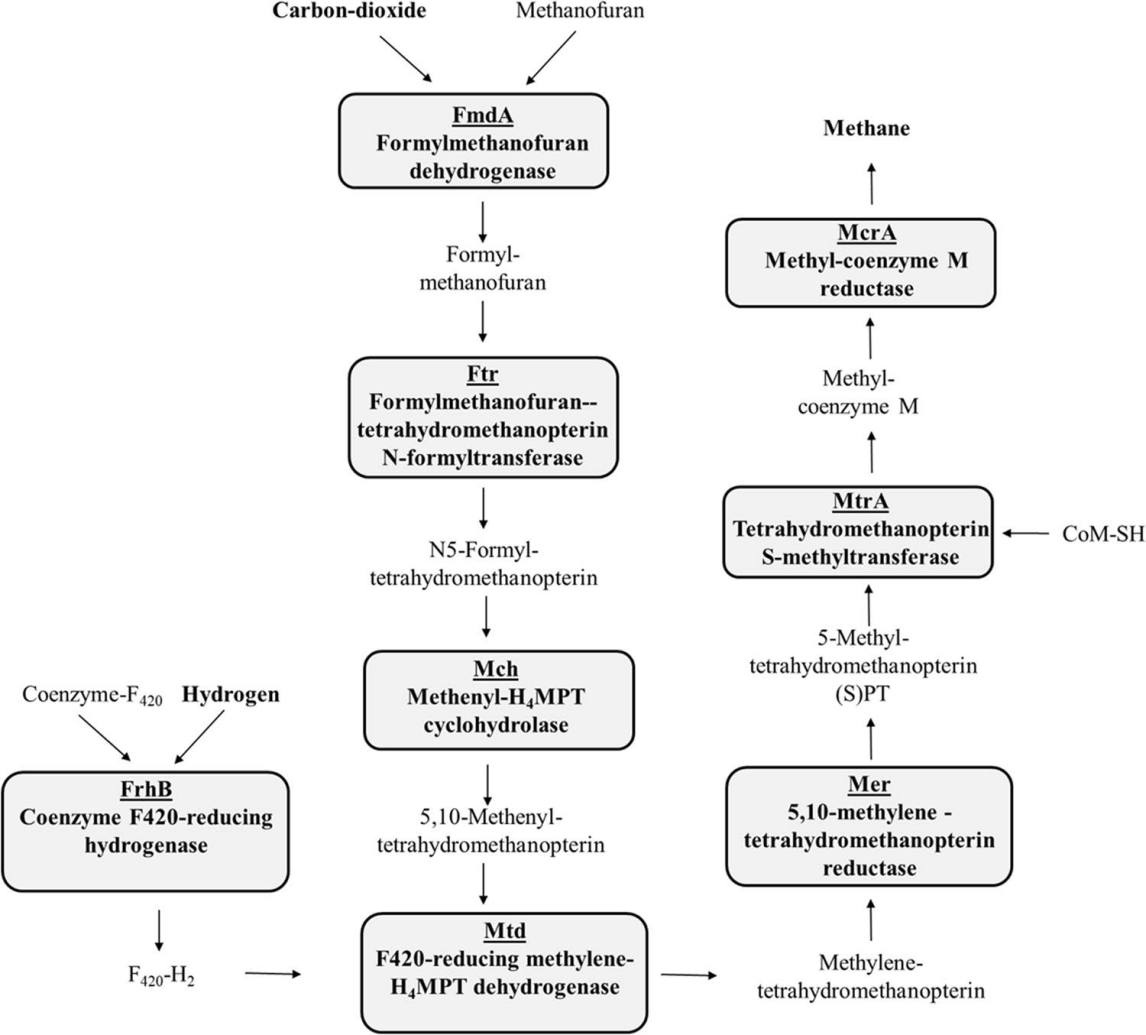
Schematic diagram of the hydrogenotrophic methanogenesis pathway

The genes of the H category regulate coenzyme transport and metabolism, so they code for methanogenesis (methyl-coenzyme M reductase (mcr)) (Thauer 1998) and the hydrogenotrophic methanogenesis-related enzyme (tetrahydromethanopterin *S*-methyltransferase (mtr)) (Wagner et al. 2018).

Previous studies demonstrated that gene expression levels in *Bacteria* reached the maximum in most cases within 1 h (Golding et al. 2005). We found in mixed enriched hydrogenotrophic cultures a similar time range (< 2–12 h) in the response to H_2_/CO_2_ addition at mesophilic conditions (Szuhaj et al. 2016; Kakuk et al. 2021) limited by the solubility of H_2_ in the aqueous system. In line with these observations, in the present study, the absence of H_2_/CO_2_ resulted in fading gene expression along the whole genome of the *M. fervens* (domain Archaea) in less than 1 h, which is only an upper time limit we could reach in this system. This is an important message for practical power-to-gas renewable electricity conversion and storage systems as in real-life P2G operation, and the biological component is not a rate-limiting component in most cases. The results thus confirm that hydrogenotrophic methanogens are effective, flexible, and easily manageable biological agents to convert the intermittently produced renewable electricity, generated by photovoltaics and/or wind power, to storable and transportable biomethane. In addition, we know from earlier studies (Szuhaj et al. 2016) that the microbial community can hibernate and inactivate itself for an extended period of time when not needed to perform. Taken together, these observations offer additional possibilities for the development of optimized microbiological communities for the P2G industry.

## Data Availability

The raw metagenome and transcriptome sequences generated and analyzed during the current study were deposited in NCBI under the bioproject accession numbers: PRJNA922065.
